# Emergent informal use of doxycycline post- and pre-exposure prophylaxis among men who have sex with men and transgender and gender diverse people, the Netherlands, 2024

**DOI:** 10.2807/1560-7917.ES.2025.30.26.2400707

**Published:** 2025-07-03

**Authors:** Buhari Teker, Elske Hoornenborg, Maarten F Schim van der Loeff, Anders Boyd, Janneke CM Heijne, Maria Prins, Udi Davidovich, Henry JC de Vries, Vita W Jongen

**Affiliations:** 1 Department of Infectious Diseases, Public Health Service of Amsterdam (GGD Amsterdam), Amsterdam, the Netherlands; 2 Department of Dermatology, Amsterdam UMC location University of Amsterdam, Amsterdam, the Netherlands; 3 Amsterdam Institute for Immunology & Infectious Diseases (AI&I), Amsterdam, the Netherlands; 4 Department of Internal Medicine, Amsterdam UMC location University of Amsterdam, Amsterdam, the Netherlands; 5 Amsterdam Public Health research institute (APH), Amsterdam, the Netherlands; 6 Stichting HIV monitoring, Amsterdam, the Netherlands; 7 Department of Social Psychology, University of Amsterdam, Amsterdam, the Netherlands

**Keywords:** doxycycline, pre-exposure prophylaxis, post-exposure prophylaxis, STI, prevention, antimicrobial-resistance

## Abstract

**BACKGROUND:**

Doxycycline post-exposure and pre-exposure prophylaxis (doxyPEP/PrEP) to prevent sexually transmitted infections (STIs) is not part of standard practice in the Netherlands but may be used informally (without prescription).

**AIM:**

We assessed informal use and intention to use doxyPEP/PrEP among men who have sex with men (MSM) and transgender and gender diverse people in the Netherlands.

**METHODS:**

Between 26 February and 6 May 2024, we conducted an online survey on informal doxyPEP/PrEP use in the preceding 6 months and intention to use. We assessed the determinants of recent informal use and high intention to use using risk ratio regression.

**RESULTS:**

Of the 1,633 participating individuals (96.7% MSM) 246 (15%) reported doxyPEP/PrEP use in the preceding 6 months. Informal use was more common among participants who used HIV PrEP or were living with HIV, had ≥ 15 anonymous sexual partners, engaged in chemsex, wanted to protect themselves, got advice from others to use doxyPEP/PrEP and believed doxyPEP/PrEP is safe. High intention to use (n = 1,063 (65%) participants) was more common among participants using HIV PrEP or living with HIV, who wanted to have condomless sex, who wanted to protect others from STIs and who perceived doxyPEP/PrEP as effective and safe.

**CONCLUSION:**

Although doxyPEP/PrEP is not formally recommended in the Netherlands, survey participants reported informal use and a high intention to use. These findings, combined with the global increase of antimicrobial resistance (AMR), stress the need for close monitoring and further research on the AMR effects of doxyPEP/PrEP use.

**Key public health message**

**What did you want to address in this study and why?**

Doxycycline used as post-exposure and pre-exposure prophylaxis (doxyPEP/PrEP) are novel strategies for preventing sexually transmitted infections (STIs). We assessed informal use (without prescription) and intention to use doxyPEP/PrEP among men who have sex with men (MSM) and transgender and gender diverse people in the Netherlands as there are few guidelines on its use. Informal use may contribute to antimicrobial resistance, affecting public health.

**What have we learnt from this study?**

We found that of 1,633 respondents to an online questionnaire 22% had ever used doxyPEP/PrEP informally, 15% reported recently using it and 65% had a high intention to use it. The proportion of informal doxyPEP/PrEP use has increased substantially since a 2021–2022 survey conducted in Amsterdam, the Netherlands. Informal use was associated with HIV PrEP use, sexualised drug use and the perception that doxyPEP/PrEP is safe to use.

**What are the implications of your findings for public health?**

Our findings underscore the need for guidelines on doxyPEP/PrEP use and the establishment of systems for monitoring guideline uptake and impact on AMR. Without proper oversight, there could be a rise in antimicrobial resistance.

## Introduction

Several randomised controlled studies (RCTs) comparing doxycycline post-exposure prophylaxis (doxyPEP) to placebo or standard care have shown that doxyPEP is an effective way to prevent syphilis (reduction in relative risk (RR) of 77–87%) and chlamydia (reduction in RR of 74–86%) among men who have sex with men (MSM) and transgender and gender diverse persons [1-3]. The effect on gonorrhoea seems more limited and varies between studies (reduction in RR of 33–55%) [1-4], possibly due to varying background prevalence of pre-existing tetracycline resistance in *Neisseria gonorrhoeae* [5]. Similarly, a small randomised controlled pilot study suggested that doxycycline pre-exposure prophylaxis (doxyPrEP) may also reduce the incidence of bacterial sexually transmitted infections (STIs) in MSM [4].

However, the public health implications of widespread doxyPEP/PrEP use are subject to debate. On the one hand, prescribing doxyPEP/PrEP to many sexually active individuals may lead to a substantial population-level increase in overall antibiotic consumption [6]. It could then lead to the development, selection and subsequent transmission of antibiotic-resistant pathogens, as well as modifying the expression of the gene encoding tetracycline resistance in the gut microbiome [2,7,8]. On the other hand, prescribing doxyPEP/PrEP for specific individuals, particularly those who repeatedly have had STIs, or who had several concomitant STIs, could prevent a substantial number of STIs with lower overall antibiotic use [6]. Currently, the formal use of doxyPEP (i.e. prescribed by a physician) is cautiously supported by clinical guidance in Australia, Germany and the United States (US) [9-11]. These guidelines advise that off-label prophylactic use of doxycycline can be prescribed on an individual basis after discussing the advantages and disadvantages of doxyPEP [9-11]. In contrast, in the United Kingdom (UK), the formal use of doxyPEP is not recommended, except in strictly experimental settings [12]. Similarly, in January 2025, a position statement on doxyPEP/PrEP was published for the Netherlands, stating that the use of doxyPEP/PrEP is not recommended [13]. In other countries, the use of doxyPEP is neither recommended nor included in guidelines. However, informal use of doxyPEP (e.g. purchasing online or over the counter without consulting a healthcare provider) has been reported in Germany, Spain and the US, and ranges between 7.3% and 54.6% among MSM and transgender women [14-16].

While doxyPEP/PrEP is currently not being recommended or actively promoted by healthcare professionals in the Netherlands, the extent of informal doxyPrEP/PEP use and demand remains unclear. Therefore, we aimed to assess informal use of doxycycline as either PEP or PrEP among MSM and transgender and gender diverse people in the Netherlands. We also assessed awareness of doxyPEP/PrEP and intention to use, as well as determinants of informal use and intention to use.

## Methods

### Study design and participants

We performed a cross-sectional study using a structured online survey on previous use of doxyPEP or doxyPrEP, awareness of doxyPEP/PrEP and intention to use doxyPEP/PrEP. Men who have sex with men and transgender and gender diverse persons of 18 years of age or older were eligible for inclusion.

Individuals were recruited through advertisements at the Centre for Sexual Health of the Public Health Service in Amsterdam, the Netherlands (a large expertise centre for STIs offering free of charge STI and sexual healthcare to several key populations, including MSM and transgender and gender diverse people). Additionally, individuals were recruited through online advertisement on Grindr (a dating app designed for MSM and used across the Netherlands), Meta (advertisement through Instagram, Facebook and Facebook Messenger), and specific Instagram platforms (@mantotman_nl, @prepnunl, @outtv, @csga020). As an incentive for participation, 10 gift vouchers of 50 euros each were raffled among the participants.

### Data collection

Data were collected between 26 February and 6 May 2024. The survey was developed by a multidisciplinary team at the Department of Infectious Diseases of the Public Health Services of Amsterdam and based on a previous survey [17]. The survey consisted of 43 questions and was available in Dutch and English; the full survey is included in Supplementary Material S1. Before starting the survey, participants received general information about doxyPEP/PrEP, including an explanation of what it is, its intended purpose and how it is used.

Participants were asked whether they have heard about doxyPEP/doxyPrEP before the survey as proxy for doxyPEP/PrEP awareness. Participants were then asked whether they had used doxyPEP/PrEP in the 6 months before completing the survey. If so, questions were asked relating to usage patterns, which antibiotic(s), methods of obtaining antibiotics and costs of the antibiotics. Of note, the use of doxyPEP/PrEP may also include the pre- or post-exposure prophylactic use of other antibiotics. Intention to use doxyPEP/PrEP was assessed on a 7-point Likert scale ranging from 1 (very low intention to use) to 7 (very high intention to use). Participants were also asked about their willingness to pay for doxyPEP/PrEP if it would become formally available. Predefined reasons for using doxyPEP/PrEP e.g. protecting oneself, having condomless sex, perceived risk of STI, were assessed on a 7-point Likert scale ranging from 1 (very unimportant) to 7 (very important). Additionally, participants reported on their sociodemographic information (gender, age, sexual preference, country of birth, country culturally most connected to, highest education level and employment status), sexual health (HIV status, use of oral HIV PrEP/PEP and history of bacterial STIs) and sexual behaviour in the preceding 6 months (number and type of sexual partners, chemsex (i.e. sex under the influence of crystal methamphetamine, mephedrone or gamma‐hydroxybutyrate acid (GHB)/gamma‐butyrolactone (GBL) or ketamine), sex under the influence of alcohol, sex under the influence of other drugs (excluding alcohol and drugs used in chemsex), injecting drugs during sex, group sex and sex work). Personal STI risk perception and beliefs around doxyPEP/PrEP effectiveness and safety were assessed on a 7-point Likert scale ranging from 1 (strongly disagree with statement) to 7 (strongly agree with statement).

Type of sexual partner was categorised into steady partners, known casual partners and anonymous casual partners. The term ‘steady partners’ refers to individuals with whom a perceived enduring relationship exists, irrespective of duration. The term ‘known casual partners’ refers to individuals with whom participants occasionally meet for sex but are not regarded as steady partners. The term ‘anonymous casual partners’ refers to individuals with whom a single sexual encounter occurred, including those encountered through dating apps. Individuals could indicate multiple types of sexual partners.

### Statistical analysis

We compared sociodemographic, clinical and sexual behaviour characteristics of participants who used and who did not use doxyPEP/PrEP in the past 6 months using Pearson’s chi-square or Fisher’s exact test for categorical variables and Student’s t-test and Wilcoxon rank-sum test for continuous variables. Participants who did not complete the questions related to age, gender and sexual preference were excluded from all analyses.

We separately assessed the determinants of two outcomes: (i) informal use of doxyPEP/PrEP in the 6 months prior to the survey, and (ii) high intention to use. As most responses regarding intention to use were concentrated at higher points of the Likert scale resulting in a heavily skewed distribution, intention to use was dichotomised as low/neutral intention (score 1–5) and high intention (score 6–7). We modelled the probability of informal use and high intention to use using RR regression (generalised linear model with a Poisson distribution family, log link and robust variance estimations). In the univariable analysis, we added individual covariates to the model to obtain the unadjusted prevalence ratio, comparing the prevalence of the outcome across levels of covariates, and their 95% confidence intervals (CI). We tested covariates using the Wald chi-square test. We then included all determinants from the univariable analysis with a p value < 0.20 in an initial multivariable model. Determinants were added in two steps to the multivariable model: (i) socio-demographic and sexual behavioural determinants, and (ii) reasons for using doxyPEP/PrEP and perceptions related to STI risk and the use of doxyPEP/PrEP. At each step, covariates were tested using the likelihood ratio test and those that did not significantly improve model fit were removed in a backward, stepwise selection. Covariates determined in the first step were forced in the model in the second step. We assessed multicollinearity between variables in the multivariable model using the variance inflation factor (VIF). If VIF was higher than 10, the variable was removed from the model.

We considered a p value of < 0.05 as statistically significant. Statistical analyses were performed using Stata version 17 (StataCorp, College Station, US).

## Results

### Description of the study population

Between 26 February and 6 May 2024, 2,094 individuals started the survey. Of these, 412 (19.7%) participants did not complete the questions regarding age, gender and/or sexual preference and were excluded from analysis. Furthermore, we excluded 49 (2.3%) cisgender women and heterosexual cisgender men. In total, 1,633 participants were included in the analysis: 1,579 (96.7%) MSM and 54 (3.3%) transgender and gender diverse persons. Participants had a median age of 37 years (interquartile range (IQR): 31–45) (Table 1). About half of the participants were born in the Netherlands (53.4%), 74.4% were culturally most connected to the Netherlands (i.e. individuals who identify most strongly with Dutch culture, regardless of their migration background), 62.9% had a college or university degree and 88.9% were employed. The median number of sex partners in the 6 months before the survey was 10 (IQR: 4–20). The majority of participants had steady (68.2%), known casual (79.2%) and/or anonymous (76.0%) sex partner(s). There were 152 participants (9.8%) living with HIV, and of the 1,396 participants without HIV who reported data on HIV PrEP use, 1,039 (74.3%) had used oral HIV PrEP in the previous 6 months.

**Table 1:** Sociodemographic, health and behavioural characteristics of participants who used and did not recently use doxycycline post-exposure and pre-exposure prophylaxis, the Netherlands, 26 February–6 May 2024

Characteristics	DoxyPEP/PrEP use in preceding 6 months						
	Total (n = 1,633)		No (n = 1,387)		Yes (n = 246)		p value^b^
	n^a^	%^a^	n^a^	%^a^	n^a^	%^a^	
Sociodemographic							
**Gender**							
Male	1,579	96.7	1,337	96.4	242	98.4	0.110
Transgender or gender diverse	54	3.3	50	3.6	4	1.6	
**Age (years)**							
Median (IQR)	37 (31–45)		37 (31–45)		37 (31–44)		0.712
< 35	670	41.0	573	41.3	97	39.4	0.417
35–44	552	33.8	460	33.2	92	37.4	
≥ 45	411	25.2	354	25.5	57	23.2	
**Country/continent of birth**							
Netherlands	837	53.4	712	53.6	125	52.5	0.690
Europe (excluding Netherlands and including Russia)	328	20.9	276	20.8	52	21.8	
Türkiye/Morocco	25	1.6	20	1.5	5	2.1	
Suriname/ Dutch Caribbean	28	1.8	24	1.8	4	1.7	
Africa (excluding Morocco)	43	2.8	40	3.0	3	1.3	
Asia (excluding Türkiye)	122	7.8	105	7.9	17	7.1	
Americas and Oceania (including Australia and New Zealand)	183	11.7	151	11.4	32	13.5	
**Country/continent most connected to**							
Netherlands	1,161	74.4	987	74.7	174	72.8	0.467
Europe (excluding Netherlands and including Russia)	272	17.4	227	17.2	45	18.8	
Türkiye/ Morocco	5	0.3	5	0.4	0	0.0	
Suriname/ Dutch Caribbean	12	0.8	11	0.8	1	0.4	
Africa (excluding Morocco)	12	0.8	12	0.9	0	0.0	
Asia (excluding Türkiye)	31	2.0	27	2.1	4	1.7	
Americas and Oceania (including Australia and New Zealand)	67	4.3	52	3.9	15	6.3	
**Highest education level**							
None, primary, secondary or other	582	37.1	492	36.9	90	37.7	0.832
College or university	989	62.9	840	63.1	149	62.3	
**Employment status**							
Unemployed or other^c^	175	11.1	155	11.6	20	8.4	0.139
Employed	1,396	88.9	1,177	88.4	219	91.6	
Health-related							
**HIV status**							
Negative	1,396	90.2	1,192	91.1	204	85.4	0.006
Positive	152	9.8	117	8.9	35	14.6	
**Use of HIV PrEP^d,e^**							
No	357	25.6	349	29.3	8	3.9	< 0.001
Yes	1,039	74.4	843	70.7	196	96.1	
**Previous use of HIV PEP^d,e^**							
No	1,373	98.3	1,176	98.7	197	96.6	0.030
Yes	23	1.7	16	1.3	7	3.4	
**Recent STI test^d^**							
No	185	11.8	182	13.7	3	1.3	< 0.001
Yes	1,385	88.2	1,149	86.3	236	98.7	
**History of any bacterial STI^d, f^**							
No	839	54.5	748	57.4	91	38.6	< 0.001
Yes	700	45.5	555	42.6	145	61.4	
Sexual behaviour-related							
**Number of sexual partner(s)^d^**							
Median (IQR)	10	(4–20)	10	(4–20)	19	(9–30)	< 0.001
**Chemsex^d,g^**							
No	1,011	64.4	911	68.4	100	41.8	< 0.001
Yes	559	35.6	420	31.6	139	58.2	
**Sex in combination with other drugs^d,h^**							
No	1,507	96.0	1,275	95.8	232	97.1	0.354
Yes	63	4.0	56	4.2	7	2.9	
**Injecting drugs during sex^d^**							
No	1,511	96.2	1,280	96.2	231	96.6	0.717
Yes	59	3.8	51	3.8	8	3.4	
**Group sex^d^**							
No	663	42.2	602	45.2	61	25.5	< 0.001
Yes	908	57.8	730	54.8	178	74.7	
**Sex work^d^**							
No	1,487	94.7	1,263	94.9	224	93.7	0.458
Yes	83	5.3	68	5.1	15	6.3	

Doxy: doxycycline; HIV: human immunodeficiency virus; IQR: interquartile range; PrEP: pre-exposure prophylaxis; PEP: post-exposure prophylaxis, STI: sexually transmitted infection.

Data were missing for: country/continent of birth (n = 67), country/continent most connected to (n = 66), education level (n = 62), employment status (n = 62), living with HIV (n = 85), recent STI test (n = 63), history of any bacterial STI (n = 94), number of sexual partner(s) (n = 65), sex in combination with alcohol (n = 63), chemsex (n = 63), sex in combination with other drugs (n = 63), injecting drugs during sex (n = 63), group sex (n = 62) and sex work (n = 63).

^a^ Unless otherwise stated.

^b^ p values were calculated using Pearson’s chi-square test or Fisher’s exact test (for categorical data) and Student’s t-test or Wilcoxon rank-sum test (for continuous data).

^c^ Other is defined as incapacitated, volunteer, retired or studying/in school.

^d^ In the preceding 6 months.

^e^ Among participants without HIV.

^f^ Includes gonorrhoea, chlamydia, lymphogranuloma venereum and syphilis.

^g^ Includes methamphetamine, gamma-hydroxybutyric acid (GHB)/gamma-butyrolactone (GBL), mephedrone and ketamine.

^h^ Excludes methamphetamine, gamma-hydroxybutyric acid (GHB)/gamma-butyrolactone (GBL), mephedrone, ketamine and alcohol.

### Informal use of doxycycline post-exposure and pre-exposure prophylaxis

Of the 1,633 participants, 368 (22.5%) reported they had ever used doxyPEP/PrEP and 246 (15.1%) reported using doxyPEP/PrEP in the 6 months prior to the survey. Participants who reported recent use of doxyPEP/PrEP were more likely to live with HIV or more frequently used oral HIV PrEP in the past 6 months compared with participants who had not used doxyPEP/PrEP (Table 1). They also more often had a history of bacterial STIs, reported a higher number of sexual partners and had more often engaged in chemsex and group sex in the past 6 months.

Of the 246 participants reporting recent doxyPEP/PrEP use, 114 (46.3%) used it as PEP, 71 (28.9%) as PrEP and 61 (24.8%) as a combination of both (Table 2). The largest proportion (40.7%) used doxyPEP/PrEP 2–4 times in the past 6 months during a median of 2 (IQR: 1–4) consecutive days. Doxycycline was the most commonly used antibiotic, while the prophylactic use of other antibiotics, including azithromycin, ciprofloxacin and amoxicillin, was also reported (Table 2). Thirty-eight participants (15.5%) did not remember which antibiotic they used for PEP/PrEP. Doxycycline PEP/PrEP was mostly used in association with anal sex (57.3%) and primarily obtained from countries outside the Netherlands or through prescription for other indications. Participants bought a median of 30 antibiotic pills (IQR: 14–60) during their most recent personal purchase and paid a median price of 30 euros (IQR: 15–50) for their pills.

**Table 2:** Information related to informal use of doxycycline post-exposure and pre-exposure in the preceding 6 months, among men who have sex with men and transgender and gender diverse persons, the Netherlands, 26 February–6 May 2024

Information related to use	DoxyPEP/PrEP use in preceding 6 months (n = 246)	
	n^a^	%^a^
Manner of use^b^		
PEP	114	46.3
PrEP	71	28.9
PEP and PrEP	61	24.8
Timing of use^b^		
In the last 3 months	194	78.9
4 to 6 months ago	52	21.1
Frequency of use^b^		
Once	56	23.2
2–4 times	100	40.7
5–10 times	36	14.6
10 or more times	53	21.5
Antibiotic used^b,c^		
Doxycycline	187	76.0
Azithromycin	30	12.2
Erythromycin	9	3.7
Ciprofloxacin	12	4.9
Amoxicillin	9	3.7
Cefixime	5	2.0
Flucloxacillin	1	0.4
Pheneticillin	1	0.4
Tetracycline	3	1.2
Unknown	38	15.5
Days used consecutively^b^		
Median (IQR)	2 (1–4)	
Use with oral sex^b,d^		
Always or mostly yes	70	28.9
Use with vaginal sex^b,e^		
Always or mostly yes	19	26.8
Use with anal sex^b,f^		
Always or mostly yes	141	57.3
Obtained through^b,c^		
Abroad	82	33.3
Prescription for other indication	77	31.3
Internet	48	19.5
Friends	47	19.1
In study context	17	6.9
Steady and casual partner(s)	16	6.5
Left over from STI treatment	13	3.3
Other (e.g. drug dealers)	3	1.2
Number of antibiotic pills obtained last time		
Median (IQR)	30 (14–60)	
Median price paid in euros (IQR)	30 (15–50)	
Pills not personally purchased/received for free	68 27.6%	

Doxy: doxycycline; IQR: interquartile range; PEP: post-exposure prophylaxis; PrEP: pre-exposure prophylaxis; STI: sexually transmitted infection.

Data were missing for: days subsequently used (n = 37), use with vaginal sex (n = 1), use with anal sex (n = 1), obtained through (n = 5), number of antibiotic pills bought (n = 1).

^a^ Unless otherwise stated.

^b^ In the preceding 6 months.

^c^ Participants could indicate multiple responses.

^d^ Among those reporting oral sex (n = 242).

^e^ Among those reporting vaginal sex (n = 71).

^f^ Among those reporting anal sex (n = 246).

Univariable determinants of recent informal doxyPEP/PrEP use can be found in Supplementary Table S1. In the multivariable analysis (Table 3), the prevalence for informal use of doxyPEP/PrEP was higher for participants who used oral HIV PrEP (p < 0.001) or were living with HIV (p < 0.001), had 15 or more anonymous sexual partners (p = 0.004), engaged in chemsex (p < 0.001), reported wanting to protect oneself (p = 0.019), got advice from others to use doxyPEP/PrEP (p = 0.003) and believed that use of doxyPEP/PrEP is safe (p = 0.003). The prevalence was lower among those worried about long-term side effects (p = 0.001).

**Table 3:** Multivariable risk ratio regression analyses of recent informal use of and high intention to use doxycycline post-exposure and pre-exposure determinants among men who have sex with men and transgender and gender diverse persons, the Netherlands, 26 February–6 May 2024

Determinants	Informal doxyPEP/PrEP use in preceding 6 months Multivariable RR regression			High intention-to-use DoxyPEP/PrEP Multivariable RR regression		
	aPR	95% CI	p value	aPR	95% CI	p value
Demographics and sexual behaviour						
**Country/continent of birth**						
The Netherlands	NA			Reference		
Other countries				1.15	1.08–1.24	< 0.001
**Employment status**						
Unemployed or other^a^	NA			Reference		
Employed				1.22	1.07–1.40	0.004
**HIV status and PrEP use**						
HIV-negative and not using HIV PrEP	Reference			Reference		
HIV-negative and using HIV PrEP	5.28	2.63–10.60	< 0.001	1.31	1.14–1.49	< 0.001
Living with HIV	5.61	2.66–11.83	< 0.001	1.30	1.11–1.52	0.001
**History of any bacterial STI^b,c^**						
No	NA			Reference		
Yes				1.06	0.99–1.14	0.080
**Consistency of condom use with anonymous sex partners^b^**						
Inconsistent	NA			Reference		
Consistent				0.89	0.77–1.04	0.134
**Number of steady sexual partners^b,d^**						
0–1 partners	Reference			NA		
≥ 2 partners	1.23	0.98–1.55	0.076			
**Number of anonymous sexual partners^b,e^**						
0–1 partners	Reference			NA		
2–5 partners	1.19	0.79–1.80	0.409			
6–14 partners	1.25	0.84–1.87	0.277			
≥ 15 partners	1.73	1.19–2.51	0.004			
**Chemsex^b,f^**						
No	Reference			NA		
Yes	1.71	1.34–2.17	< 0.001			
**Group sex^b^**						
No	NA			Reference		
Yes				1.08	1.00–1.16	0.064
Reasons for using/not using DoxyPEP/PrEP						
**Self-protection^g^**						
Low/neutral	Reference			NA		
High	2.66	1.17–6.04	0.019			
**Protection of others^g^**						
Low/neutral	NA			Reference		
High				1.19	1.06–1.32	0.002
**To have condomless sex^g^**						
Low/neutral	NA			Reference		
High				1.10	1.001–1.22	0.047
**Considered oneself at high risk for STIs^g^**						
Low/neutral	NA			Reference		
High				1.18	1.09–1.28	< 0.001
**Advice of others^g^**						
Low/neutral	Reference			Reference		
High	1.51	1.15–1.98	0.003	1.11	1.03–1.20	0.005
**Worried about short-term side effects^g^**						
Low/neutral	NA			Reference		
High				0.90	0.83–0.97	0.008
**Worried about long-term side effects^g^**						
Low/neutral	Reference			NA		
High	0.65	0.50–0.84	0.001			
Perceptions on STI risk, doxyPEP/PrEP effectiveness and safety						
**Generally being worried about contracting an STI^g^**						
Low/neutral	NA			Reference		
High				1.23	1.14–1.33	< 0.001
**Trust in effectiveness of doxyPEP/PrEP^g^**						
Low/neutral	NA			Reference		
High				1.11	1.01–1.23	0.039
**Trust in safety of doxyPEP/PrEP^g^**						
Low/neutral	Reference			Reference		
High	1.48	1.14–1.91	0.003	1.13	1.03–1.25	0.012
**Concerned of AMR due to use^g^**						
Low/neutral	NA			Reference		
High				0.85	0.79–0.91	< 0.001

AMR: antimicrobial resistance, aPR: adjusted prevalence ratio; CI: confidence interval; Doxy: doxycycline; HIV: human immunodeficiency virus; NA: not applicable; PEP: post-exposure prophylaxis; PrEP: pre-exposure prophylaxis; RR: risk ratio; STI: sexually transmitted infection.

^a^ Other is defined as incapacitated, volunteer, retired or studying/in school.

^b^ In the 6 months before completing the questionnaire.

^c^ Includes gonorrhoea, chlamydia, lymphogranuloma venereum and syphilis.

^d^ Categorised in quantiles.

^e^ Categorised in quartiles.

^f^ Includes methamphetamine, gamma-hydroxybutyric acid (GHB)/gamma-butyrolactone (GBL), mephedrone and ketamine.

^g^ Dichotomised due to skewed distribution: scores of 1 to 5 were categorised as low/neutral, and scores of 6 and 7 categorised as high.

Note: recent informal use of doxyPEP/PrEP is defined as use in the preceding 6 months.

### Awareness and intention to use doxycycline post-exposure and pre-exposure prophylaxis

Of 1,633 participants, 1,037 (63.5%) were aware of doxyPEP/PrEP and intention to use doxyPEP/PrEP was very high (median Likert-score: 7, IQR: 5–7, range: 1–7). More than half of the participants (n = 1,063, 65.1%) had a high intention to use doxyPEP/PrEP (score of 6 or 7) and 1,179 (72.2%) participants were willing to pay for doxyPEP/PrEP if it became formally available. Reasons for intention to use doxyPEP/PrEP included protection against STIs, protection of sex partner(s), reducing STI transmission, wanting to engage in condomless sex and enhancing sexual pleasure (Figure).

**Figure:**
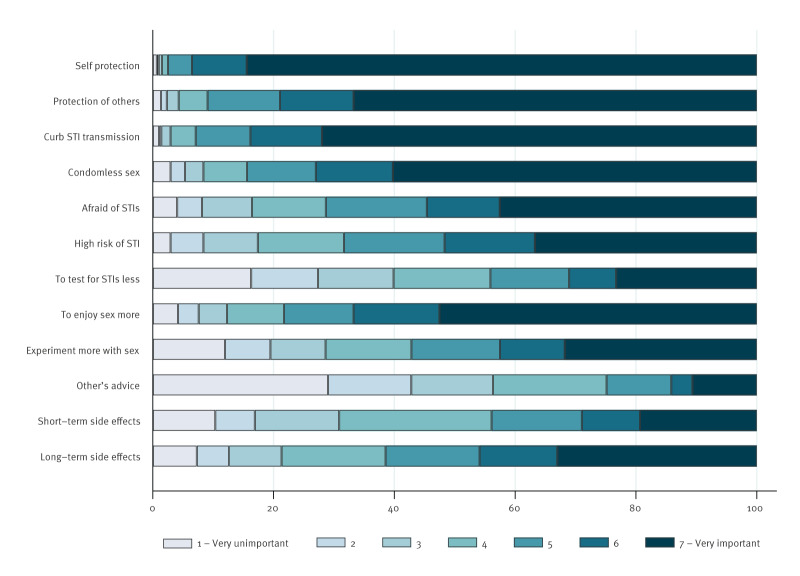
Reasons to use or not use doxycycline post-exposure and pre-exposure among men who have sex with men and transgender and gender diverse persons, the Netherlands, 26 February–6 May 2024

STI: sexually transmitted infection.

Univariable determinants of high intention to use doxyPEP/PrEP are reported in Supplementary Table S1. In the multivariable analysis (Table 3), the prevalence of having high intention to use doxyPEP/PrEP was higher among participants who were born outside the Netherlands (p < 0.001), were employed (p = 0.004), used oral HIV PrEP (p < 0.001) or were living with HIV (p = 0.001), wanted to protect others from STIs (p = 0.002), wanted to have condomless sex (p = 0.047), perceived themselves to be at risk for STIs (p < 0.001), received advice from others to use doxyPEP/PrEP (p = 0.005), were generally worried about contracting STIs (p < 0.001) or perceived doxyPEP/PrEP as effective (p = 0.039) and safe (p = 0.012). Prevalence was lower for those worried about short-term side effects (p = 0.008) or were concerned about antimicrobial resistance (AMR) due to doxyPEP/PrEP use (p < 0.001).

## Discussion

We found that 15% of participants in an online survey among MSM and transgender and gender diverse people in the Netherlands had recently used doxycycline to prevent STIs, despite it not being recommended in the current Dutch position statement on doxyPEP/PrEP [13]. Doxycycline was the most commonly used antibiotic for PEP/PrEP albeit other types of antibiotics were also used. Moreover, the majority of participants were aware of doxyPEP/PrEP and had a high intention to use. Determinants for both informal use and a high intention to use doxyPEP/PrEP included using oral HIV PrEP or living with HIV, receiving advice from others to use doxyPEP/PrEP and perceiving doxyPEP/PrEP as effective and safe.

We found that 22.5% of participants had ever used doxyPEP/PrEP and 15.1% had used doxyPEP/PrEP in the 6 months prior to the survey. These proportions were substantially higher than those found in a previous study among MSM with comparable sexual behaviour from the Amsterdam Cohort Studies, where only 2.5% of 593 participants had ever used doxyPEP in 2021–2022 [17]. This suggests a rapidly growing uptake of informal use of doxyPEP/PrEP in the Netherlands. Additionally, 29% of participants with recent doxyPEP/PrEP use reported using it specifically as doxyPrEP, which is not recommended in any clinical guidance documents [9-13]. Moreover, both awareness and intention to use doxyPEP/PrEP in our current study were substantially higher compared with the earlier survey [17]. Possible reasons include that in 2021–2022, results from the first large RCTs on doxyPEP had not yet been published [2,3], thus familiarity with doxyPEP/PrEP was much lower. Studies conducted in Germany and the US among MSM and transgender women in 2023, before the publication of the US Centers for Disease Control and Prevention (CDC) guideline and the German STI Society’s position statement on doxyPEP [9,10], also found lower informal use, awareness and intention to use doxyPEP compared with our study [14,15]. By contrast, a study conducted in Spain in 2024 found a higher proportion of respondents who had ever used doxyPEP/PrEP (54.6%) [16]. A possible explanation for the difference in our study’s results compared with the Spanish study is the much higher prevalence of sexualised drug use in the Spanish study. As individuals engaging in sexualised drug use have a higher risk of STIs [18], this may have also increased their willingness to use doxyPEP/PrEP. As clinical guidance on doxyPEP/PrEP is expected to become available in more countries or continents, awareness and intention are expected to increase.

Additionally, our study found that informal use and high intention to use doxyPEP/PrEP may be influenced by others' advice, which could lead to poorly informed decision-making due to a potential lack of knowledge about doxyPEP/PrEP and related issues, such as AMR. In combination with the relatively high proportion of participants who had ever and had recently used doxyPEP/PrEP informally, it underscores the need for guidelines, information campaigns and comprehensive individual-level explanations during healthcare visits. These are crucial to guide healthcare workers and potential or current users. After completion of our study, the Netherlands published a position statement not recommending the use of doxyPEP or doxyPrEP [13]. However, to mitigate the risks of unsupervised informal use, doxyPEP may be prescribed on an individual basis – with comprehensive counselling on its drawbacks – to those who are already using doxyPEP without medical supervision [13].

While there are advantages of doxyPEP such as reduced risk of syphilis and chlamydia, there is uncertainty about the long-term adverse effects of doxyPEP/PrEP use. Specifically, the impact of prophylactic antibiotic use on AMR needs to be taken into account. A recent study from the US on doxyPEP effectiveness and its effects on AMR found higher proportions of doxycycline-resistant *N. gonorrhoeae* in the doxyPEP group compared with the control group receiving local standard care without doxyPEP and a similar distribution of resistant strains of *Staphylococcus aureus* [2]. Additionally, doxyPEP use increased tetracycline resistance genes in the gut microbiome after 6 months of use, with higher doxycycline exposure correlating with greater resistance, although microbiome diversity remained stable [8]. If doxyPEP is implemented, it is essential to closely monitor both individual and population-level resistance to doxycycline, particularly when doxycycline is used as a first-line or alternative treatment for STIs (e.g. for *Chlamydia trachomatis* infections), alongside monitoring AMR development in various pathogens [19]. Of note, we found that 24% of participants in the present study used antibiotics other than doxycycline to prevent STIs, some of which were second-line antibiotics, while effectiveness and safety of these medications as prophylaxis against bacterial STIs have not been assessed. Therefore, the use of antibiotics other than doxycycline should be strongly discouraged, as is also stated, for example, in the Australian consensus statement regarding doxyPEP/PrEP and Dutch position statement on doxyPEP/PrEP [9-11,13].

We found several factors associated with informal use and a high intention to use doxyPEP/PrEP. These factors included sexual behaviour associated with increased STI risk, self-perceived higher risk of STIs, generally being worried about contracting an STI, wanting to protect oneself or others and wanting to have condomless sex. Furthermore, participants using oral HIV PrEP or living with HIV were more likely to use doxyPEP/PrEP or have a high intention to use it. These findings align with previous studies indicating a correlation between HIV PrEP usage and the use of doxyPEP [14,20]. However, a recent study suggested that the use of HIV PrEP or living with HIV may not be a good indicator for doxyPEP prescription and that prescribing strategies based on STI history may be more efficient and may prevent more STI diagnoses than those based on HIV PrEP use or HIV status [6]. Targeting a smaller, well-defined subpopulation with a high incidence of STIs could help limit the increase in antibiotic consumption at the population level, while still effectively reducing the incidence of bacterial STIs [21]

This study is not without limitations. First, our participants were primarily MSM, highly educated, born in the Netherlands and did not live with HIV. Moreover, the median number of sexual partners in the previous 6 months was relatively high. Therefore, they may not be representative of transgender and gender diverse people or the broader MSM population. Second, despite providing comprehensive explanations about the topic, misunderstandings could still arise due to the relatively new nature of the subject, for example confusion between doxyPEP/PrEP use and oral HIV PEP or PrEP use. Third, participants may have under-reported their sexual behaviour as they may believe it is perceived as undesirable [22].

## Conclusions

While doxyPEP/PrEP can prevent STIs, it is currently not recommended through routine healthcare settings in the Netherlands. Still, it was recently used informally by 15% of participants in our study. Moreover, the majority of participants were aware of doxyPEP/PrEP and intention to use was very high. Lack of monitoring and regulation of doxyPEP/PrEP makes it difficult to detect overuse, misuse and adverse effects including AMR development and effects on microbiome composition. Thus, close monitoring and further research on AMR effects of doxyPEP/PrEP are needed.

## Data Availability

Data are available upon reasonable request. On reasonable request to the last author (vjongen@ggd.amsterdam.nl), the following data will be made available after publication: de-identified participant data. Data will be shared after approval of an analysis proposal by the co-authors (BT, MSvdL, AB, EH, UD, JH, MP, HdV, VWJ).

## Supplementary Data

329000 Supplementary Material
