# Supplementary material for: Emergent informal use of doxycycline post- and pre-exposure prophylaxis among men who have sex with men and transgender and gender diverse people, the Netherlands, 2024
Source: Euro Surveill. 2025 Jul 3;30(26):2400707. doi: 10.2807/1560-7917.ES.2025.30.26.2400707 (PMC12231377; doi:10.2807/1560-7917.ES.2025.30.26.2400707)
Supplement: Supplementary Material [file 24-00707_TEKER_Supplement.pdf]

SUPPLEMENT TO:

Emergent informal use of doxycycline post- and pre-exposure prophylaxis among men who have sex with men and transgender and gender diverse people, the Netherlands, 2024

This supplementary material is hosted by *Eurosurveillance* as supporting information alongside the article "Emergent informal use of doxycycline post- and pre-exposure prophylaxis among men who have sex with men and transgender and gender diverse people, the Netherlands, 2024", on behalf of the authors, who remain responsible for the accuracy and appropriateness of the content. The same standards for ethics, copyright, attributions and permissions as for the article apply. Supplements are not edited by *Eurosurveillance* and the journal is not responsible for the maintenance of any links or email addresses provided therein.

Supplementary Material S1: Survey to assess informal use and intention-to-use of doxyPEP/doxyPrEP, the Netherlands, 26 February 2024 to 6 May 2024

1. What is your gender (as registered at birth)?
   - ☐ Female
   - ☐ Male
   - ☐ Intersex:
     - ☐ registered as female
     - ☐ registered as male

2. What is your gender identity:
   - ☐ Female
   - ☐ Male
   - ☐ Other, namely…… (non-binary, genderfluid, agender, etc.)

3. Who are you attracted to? (romantically and/or sexually)
   (Multiple answers are possible; check all options that apply)
   - ☐ Women
   - ☐ Men
   - ☐ Other gender identities, namely:
     - ☐ Transgender women
     - ☐ Transgender men
     - ☐ Gender diverse people (non-binary, genderfluid, agender, etc.)

4. What is your age? ____ years

The following questions relate to PrEP/PEP for STIs

PrEP (pre-exposure prophylaxis) for STIs is the preventive use of antibiotics **before** sexual activity to prevent STIs (chlamydia, gonorrhea, and syphilis), while **PEP (post-exposure prophylaxis)** is the preventive use of antibiotics **after** sexual activity to prevent STIs. PrEP/PEP for STIs is different from PrEP/PEP for HIV or STI treatment.

5. Did you know of PrEP/PEP for STIs before this survey?
   ☐ No | ☐ Yes

6. Did you ever use antibiotics to prevent STIs (PrEP/PEP for STIs)?
   - ☐ No
   - ☐ Yes, as PrEP
   - ☐ Yes, as PEP
   - ☐ Yes, both as PrEP and PEP

7. Did you use PrEP/PEP for STIs in the previous 6 months?
   - ☐ No
   - ☐ Yes, in the previous 3 months
     - ☐ As PrEP
     - ☐ As PEP
     - ☐ Both as PrEP and PEP
   - ☐ Yes, in the previous 4-6 months
     - ☐ As PrEP
     - ☐ As PEP
     - ☐ Both as PrEP and PEP

8. How often did you use PrEP/PEP for STIs in the previous 6 months?
   - ☐ Once
   - ☐ 2 to 4 times
   - ☐ 5 to 10 times
   - ☐ More than 10 times

9. Which antibiotic did you use? (multiple answers are possible)
   - ☐ Doxycycline
   - ☐ Azithromycin (Zithromax)
   - ☐ Erythromycin (Erythrocine)
   - ☐ Ciprofloxacin
   - ☐ Amoxicillin (Augmentin)
   - ☐ Cefixime
   - ☐ Flucloxacillin (Floxapen)
   - ☐ Pheneticillin (Broxil)
   - ☐ Tetracycline
   - ☐ Other, namely:
   - ☐ I do not know

10. How many consecutive days did you use PrEP/PEP for STIs? If you used PrEP/PEP for STIs multiple times in the previous 6 months, please think about the last time you used it.

    ……… day(s)
    - ☐ I do not know

11. How often did you use PrEP/PEP for STIs because you had oral sex ("blowjob") without a condom in the previous 6 months?
    - ☐ I did not have oral sex
    - ☐ Never
    - ☐ Usually not
    - ☐ Sometimes yes – sometimes no
    - ☐ Most of the time
    - ☐ Always

12. How often did you use PrEP/PEP for STIs because you had anal sex without a condom in the previous 6 months?
    - ☐ I did not have anal sex
    - ☐ Never
    - ☐ Usually not
    - ☐ Sometimes yes – sometimes no
    - ☐ Most of the time
    - ☐ Always

13. How often did you use PrEP/PEP for STIs because you had vaginal sex without a condom in the previous 6 months?
    - ☐ I did not have vaginal sex
    - ☐ Never
    - ☐ Usually not
    - ☐ Sometimes yes – sometimes no
    - ☐ Most of the time
    - ☐ Always

14. How did you obtain PrEP/PEP for STIs (multiple answers possible)?
    - ☐ Through my steady partner
    - ☐ Through my casual sex partner(s)
    - ☐ Through friends
    - ☐ Ordered through the internet
    - ☐ Bought outside of the Netherlands
    - ☐ Prescribed by a doctor
    - ☐ Through participation in a study on PrEP/PEP for STIs (in a study setting)
    - ☐ Other (such as through acquittances, drug dealers, etc.): _________

15. How many pills did you buy the last time you bought PrEP/PEP for STIs?
    _______ pills

16. How much did you pay for those pills?
    _______ euro
    ☐ I paid in a different currency, namely: <u>(fill in the amount here)</u> <u>(fill in the currency here)</u>

17. Do you know anyone who uses PrEP/PEP for STIs? (multiple answers possible)
    - ☐ No
    - ☐ Yes, steady partner(s)
    - ☐ Yes, casual partner(s)
    - ☐ Yes, friends
    - ☐ Yes, acquittances
    - ☐ Yes, other

18. Do you plan on using PrEP/PEP for STIs (again) in the future?
    ☐ No | ☐ Yes | ☐ I do not know

19. How likely would you start using PrEP/PEP for STIs (again) in the future?
    Very unlikely 1 2 3 4 5 6 7 Very likely

20. If PrEP/PEP for STIs were to become available in the Netherlands, where would you want to obtain it from?

☐ GGD/Center for Sexual Health/Public Health Service	☐ GP/family doctor	☐ Medical specialist	☐ Other health care provider, namely …

21. If PrEP/PEP for STIs would become available free-of-charge in the Netherlands (e.g., through general practitioners/family doctor or the Centre of Sexual Health in your region), then I would use PrEP/PEP for STIs.
    Completely disagree 1 2 3 4 5 6 7 Completely agree

22. Are you willing to pay money for the use of PrEP/PEP for STIs?
    ☐ No | ☐ Yes

23. What is the maximum amount of money you would be willing to pay per month for PrEP/PEP for STIs?
    - ☐ 0 – 5 euros
    - ☐ 6 – 10 euros
    - ☐ 11 – 15 euros
    - ☐ 16 – 20 euros

☐ More than 20 euros

24. Please indicate how important the following reasons are for you in the decision to use PrEP/PEP for STIs.

a. To protect myself against STI
Very unimportant 1 2 3 4 5 6 7 Very important

b. To protect others against STIs
Very unimportant 1 2 3 4 5 6 7 Very important

c. To reduce the spread of STIs
Very unimportant 1 2 3 4 5 6 7 Very important

d. To be able to have sex without a condom without contracting an STI
Very unimportant 1 2 3 4 5 6 7 Very important

e. Because I am anxious of getting infected with an STI
Very unimportant 1 2 3 4 5 6 7 Very important

f. Because I am often at risk of getting infected with an STI
Very unimportant 1 2 3 4 5 6 7 Very important

g. Because I can get tested for STIs less often
Very unimportant 1 2 3 4 5 6 7 Very important

h. To be able to enjoy sex more
Very unimportant 1 2 3 4 5 6 7 Very important

i. To experiment with sex
Very unimportant 1 2 3 4 5 6 7 Very important

j. Because people close to me advised me to use PrEP/PEP for STIs
Very unimportant 1 2 3 4 5 6 7 Very important

k. The chance of developing side effects immediately or shortly after using PrEP/PEP for STI
Very unimportant 1 2 3 4 5 6 7 Very important

l. The chance of developing long-term side effects after using PrEP/PEP for STI
Very unimportant 1 2 3 4 5 6 7 Very important

Indicate to what extent you agree with the following five statements.

25. I am worried about getting an STI
Strongly disagree 1 2 3 4 5 6 7 Strongly agree

26. It is awful when I get an STI
Strongly disagree 1 2 3 4 5 6 7 Strongly agree

27. If I use PrEP/PEP for STIs, I am concerned about antibiotic resistance of bacteria that cause STIs.

(Antibiotic resistance means antibiotics do not work anymore against bacterial infections because the bacteria have become immune to them.)

☐ Strongly disagree 1 2 3 4 5 6 7 Strongly agree
☐ I do not know

28. I am confident in the effectiveness of PEP/PrEP

Strongly disagree 1 2 3 4 5 6 7 Strongly agree

29. I am confident in the safety of PEP/PrEP

Strongly disagree 1 2 3 4 5 6 7 Strongly agree

Thank you for completing the questions about PEP/PrEP for STIs. We would like to ask you a few more general questions.

30. In which country/region/continent were you born?

☐ The Netherlands
☐ Europe (**excluding** the Netherlands and including Russia)
☐ Türkiye (formerly known as Turkey)
☐ Morocco
☐ Suriname
☐ Dutch Caribbean
☐ Indonesia
☐ Africa (**excluding** Morocco)
☐ Asia (**excluding** Türkiye and Indonesia)
☐ America and Oceania (**including** Australia and New Zealand)
☐ Other, namely......

31. With which country/region/continent do you feel the most connected?

☐ The Netherlands
☐ Europe (**excluding** the Netherlands and including Russia)
☐ Türkiye (formerly known as Turkey)
☐ Morocco
☐ Suriname
☐ Dutch Caribbean
☐ Indonesia
☐ Africa (**excluding** Morocco)
☐ Asia (**excluding** Türkiye and Indonesia)
☐ America and Oceania (**including** Australia and New Zealand)
☐ Other, namely......

32. What is the highest educational level you finished or that you are currently completing?
    a. Primary school
    b. High/secondary school
    c. College
    d. University
    e. Other, namely….

33. Do you currently hold a paying job? (Note: Being on sick leave is considered paid work)

a. No, I am studying/in school.

b. No, I work as a volunteer.

c. No, I am unemployed.

d. No, I am incapacitated.

e. No, I am retired.

f. Yes, I work full-time (36 hours per week or more).

g. Yes, I work part-time (less than 36 hours a week) (and combine that with my study/school)

34. Did you receive money or gifts for sex in the previous 6 months?
    a. No
    b. Yes

35. What is your HIV-status

☐ Negative
☐ Positive
☐ I do not know
☐ I do not want to say

36. Did you use pre-exposure prophylaxis (PrEP) or post-exposure prophylaxis (PEP) for **HIV** in the previous 6 months?
    a. No
    b. Yes, PrEP for HIV
    c. Yes, PEP for HIV
    d. Yes, both PEP and PrEP for HIV

37. Have you been tested for an STI in the previous 6 months, and if so, how often?
    a. No
    b. Yes, once
    c. Yes, 2-3 times
    d. Yes, 4 or more times
    e. I do not know

38. Were you diagnosed with a bacterial STI (e.g., chlamydia, gonorrhea or syphilis) in the previous 6 months?
    a. No
    b. Yes
    c. I do not know

39. With how many different sex partners did you have anal and/or vaginal sex in the previous 6 months?_______ (*letters not allowed, numbers only)*

40. *With how many steady partners, known casual partner(s), or anonymous casual partner(s) did you have anal and/or vaginal sex in the previous 6 months? (only numbers are allowed)*

Known casual partner: *with a known casual partner we mean someone you know, of whom you have contact details and with whom you sometimes meet for sex, but whom you do not see as a steady partner (e.g. sex buddy).*

Anonymous casual partner(s): *with an unknown casual partner we mean someone you do not know. Sexual partners met through Grindr fall under this category if you have not met this person before (e.g. a one night stand)*

a. Steady partner(s): ______

aa. Did you use a condom during the anal and/or vaginal sex? If the condom broke, use "sometimes"

☐ No, never | ☐ Yes, always | ☐ Sometimes

b. Known casual partner(s): ______
bb. Did you use a condom during the anal and/or vaginal sex? If the condom broke, use "sometimes"

☐ No, never | ☐ Yes, always | ☐ Sometimes

c. Anonymous casual partner(s): ______
cc. Did you use a condom during the anal and/or vaginal sex? If the condom broke, use "sometimes"

☐ No, never | ☐ Yes, always | ☐ Sometimes

41. Did you use any of the following substances during or before sex in the previous 6 months? (multiple answers possible)

    a. No
    b. Yes, namely:
        i. Alcohol
        ii. Hash/weed
        iii. Amphetamines (speed, pep)
        iv. Cocaine
        v. GHB/GBL
        vi. Ketamine (Special K)
        vii. Mephedrone (Miauw Miauw/4MMC/3MMC/4MEC)
        viii. Poppers
        ix. XTC/MDMA
        x. 4FA/4FMP
        xi. 2CB
        xii. Erectile disfunction drugs (Viagra, Sildenafil, Tadalafil, Cialis, Vardenafil, Levrita, Kamagra, Uprima)
        xiii. Methylamphetamine (crystal meth, tina, ice, crank, yabba, glass, quartz, perf, ventana)
        xiv. LSD (acid)
        xv. Heroin
        xvi. Other, namely...

42. Did you inject drugs before or during sex in the previous 6 months (slamming)?
    a. No
    b. Yes

43. Did you participate in group sex in the previous 6 months (sex with multiple individuals simultaneously or shortly after one another)?
    a. No
    b. Yes

Supplementary Table S1: Determinants of informal use of doxyPEP/doxyPrEP and high intention to use doxyPEP/PrEP. Results of univariable relative risk regression analyses, the Netherlands, 26 February 2024 to 6 May 2024

	Used informally *n (%)*[1]	**Informal doxyPEP/PrEP use in preceding 6 months** Univariable relative risk regression *PR*	*95% CI*	*p-value*	High intention *n (%)*[2]	**High intention to use DoxyPEP/PrEP** Univariable relative risk regression *PR*	*95% CI*	*p-value*
Demographics & sexual behaviour								
**Age (years)**								
<35	97 (39.4%)	REF			410 (38.6%)	REF		
35-44	92 (37.4%)	1.15	0.89-1.50	0.292	388 (36.5%)	1.15	1.06-1.25	0.001
≥45	57 (23.2%)	0.96	0.71-1.30	0.781	265 (24.9%)	1.05	0.96-1.16	0.275
**Country/region of birth**								
Netherlands	125 (52.5%)	REF			481 (46.8%)	REF		
Other countries	113 (47.5%)	1.04	0.79-1.38	0.756	547 (53.2%)	1.31	1.22-1.40	<0.001
**Country/region most connected to**								
Netherlands	174 (72.8%)	REF			760 (73.9%)	REF		
Other countries	65 (27.2%)	1.07	0.82-1.39	0.621	269 (26.1%)	1.01	0.93-1.10	0.770
**Highest education level**								
None, primary, secondary, or other	90 (37.7%)	REF			378 (36.7%)	REF		
College and university	149 (62.3%)	0.97	0.77-1.24	0.832	653 (63.3%)	1.02	0.94-1.10	0.665
**Employment status**								
Unemployed or other[3]	20 (8.4%)	REF			97 (9.4%)	REF		
Employed	219 (91.6%)	1.37	0.89-2.11	0.149	934 (90.6%)	1.21	1.05-1.39	0.007
**HIV status & PrEP use**								
HIV-negative and not using PrEP	8 (3.4%)	REF			162 (16.0%)	REF		
HIV-negative and using PrEP	193 (81.8%)	8.33	4.15-16.72	<0.001	733 (72.3%)	1.56	1.39-1.76	<0.001
Living with HIV	35 (14.8%)	10.28	4.88-21.63	<0.001	119 (11.7%))	1.73	1.50-1.99	<0.001
**History of any bacterial STI[4,5]**								
No	91 (38.6%)	REF			478 (47.3%)	REF		
Yes	145 (61.4%)	1.91	1.50-2.43	<0.001	532 (52.7%)	1.33	1.24-1.43	<0.001
**No. of steady sexual partners[4,6]**								
0-1 partners	147 (62.3%)	REF			698 (69.6%)	REF		
≥ 2 partners	89 (37.7%)	1.58	1.24-2.00	<0.001	305 (30.4%)	1.14	1.06-1.22	0.001

**No. of known casual sexual partners**[4,7]								
0-1 partners	52 (22.1%)	REF			275 (27.4%)	REF		
2-3 partners	51 (21.7%)	1.10	0.76-1.58	0.612	271 (27.1%)	1.10	1.00-1.22	0.061
4-5 partners	42 (17.9%)	1.46	1.004-2.14	0.047	182 (18.2%)	1.20	1.08-1.34	<0.001
≥6 partners	90 (38.3%)	2.22	1.62-3.03	<0.001	274 (27.3%)	1.28	1.16-1.40	<0.001
**No. of anonymous sexual partners**[4,7]								
0-1 partners	33 (14.0%)	REF			222 (22.1%)	REF		
2-5 partners	49 (20.8%)	1.71	1.13-2.61	0.012	248 (24.7%)	1.29	1.15-1.45	<0001
6-14 partners	59 (25.0%)	2.08	1.39-3.11	<0.001	261 (26.0%)	1.37	1.23-1.53	<0.001
≥ 15 partners	95 (40.3%)	3.28	2.26-4.75	<0.001	274 (27.3%)	1.41	1.26-1.57	<0.001
**Consistency of condom use with steady sex partners**								
Inconsistent	201 (96.6%)	REF			832 (95.5%)	REF		
Consistent	7 (3.4%)	0.62	0.30-1.27	0.190	39 (4.5%)	0.84	0.68-1.03	0.091
**Consistency of condom use with steady known casual partners**								
Inconsistent	205 (93.2%)	REF			851 (91.1%)	REF		
Consistent	15 (6.8%)	0.62	0.38-1.01	0.057	83 (8.9%)	0.82	0.71-0.95	0.009
**Consistency of condom use with anonymous casual partners**								
Inconsistent	208 (95.0%)	REF			811 (89.6%)	REF		
Consistent	11 (5.0%)	0.32	0.18-0.58	<0.001	94 (10.4%)	0.70	0.61-0.81	<0.001
**Chemsex**[4,8]								
No	100 (41.8%)	REF			617 (59.8%)	REF		
Yes	139 (58.2%)	2.51	1.99-3.18	<0.001	414 (40.2%)	1.21	1.13-1.30	<0.001
**Sex in combination with alcohol**[4]								
No	108 (45.2%)	REF			458 (44.4%)	REF		
Yes	131 (54.8%)	1.01	0.80-1.28	0.905	573 (55.6%)	1.05	0.97-1.12	0.221
**Sex in combination with other drugs**[4]								
No	232 (97.1%)	REF			992 (96.2%)	REF		
Yes	7 (2.9%)	0.72	0.35-1.47	0.367	39 (3.8%)	0.94	0.77-1.15	0.541
**Injecting drugs during sex**[4]								
No	231 (96.7%)	REF			986 (95.6%)	REF		
Yes	8 (3.4%)	0.89	0.46-1.71	0.720	45 (4.4%)	1.17	1.01-1.35	0.038
**Group sex**[4]								
No	61 (25.5%)	REF			371 (36.0%)	REF		

Yes	178 (74.5%)	2.13	1.62-2.80	<0.001	660 (64.0%)	1.30	1.20-1.40	<0.001
**Sex work[4]**								
No	224 (93.7%)	REF			966 (93.7%)	REF		
Yes	15 (6.3%)	1.20	0.75-1.93	0.451	65 (6.3%)	1.21	1.07-1.36	0.002
Reasons for using/not using DoxyPEP/PrEP								
**Self-protection[9]**								
Low/neutral	5 (2.0%)	REF			34 (3.2%)	REF		
High	241 (98.0%)	3.35	1.41-7.94	0.006	1,029 (96.8%)	2.10	1.59-2.78	<0.001
**Protection of others[9]**								
Low/neutral	51 (20.7%)	REF			163 (15.3%)	REF		
High	195 (79.3%)	1.02	0.77-1.36	0.889	900 (84.7%)	1.47	1.31-1.66	<0.001
**Contribute to curbing STI transmission[9]**								
Low/neutral	44 (17.9%)	REF			113 (10.6%)	REF		
High	202 (82.1%)	0.89	0.66-1.19	0.426	959 (89.4%)	1.62	1.41-1.87	<0.001
**To have condomless sex[9]**								
Low/neutral	43 (17.5%)	REF			225 (21.2%)	REF		
High	203 (82.5%)	1.75	1.28-2.38	<0.001	838 (78.8%)	1.38	1.25-1.52	<0.001
**Being afraid of contracting an STI[9]**								
Low/neutral	103 (41.9%)	REF			442 (41.6%)	REF		
High	143 (58.1%)	1.15	0.91-1.46	0.232	621 (58.4%)	1.17	1.08-1.26	<0.001
**Considered oneself at high risk for STIs[9]**								
Low/neutral	87 (35.4%)	REF			412 (38.8%)	REF		
High	159 (64.6%)	1.71	1.34-2.18	<0.001	651 (61.2%)	1.48	1.37-1.59	<0.001
**To test less for STIs[9]**								
Low/neutral	171 (69.5%)	REF			675 (63.5%)	REF		
High	75 (30.5%)	0.97	0.76-1.25	0.837	388 (36.5%)	1.28	1.19-1.37	<0.001
**To enjoy sex more[9]**								
Low/neutral	75 (30.5%)	REF			287 (27.0%)	REF		
High	171 (69.5%)	1.14	0.88-1.46	0.320	776 (73.0%)	1.35	1.23-1.47	<0.001
**To experiment more with sex[9]**								
Low/neutral	139 (56.5%)	REF			563 (53.0%)	REF		
High	107 (43.5%)	1.04	0.82-1.31	0.738	399 (47.0%)	1.20	1.12-1.28	<0.001
**Advice of others[9]**								
Low/neutral	190 (77.2%)	REF			862 (81.1%)	REF		

High	56 (22.8%)	1.79	1.37-2.33	<0.001	201 (18.9%)	1.42	1.33-1.51	<0.001
**Worried of short-term side effects[9]**								
Low/neutral	187 (76.0%)	REF			774 (72.8%)	REF		
High	59 (24.0%)	0.77	0.59-1.02	0.065	289 (27.2%)	0.92	0.84-0.99	0.037
**Worried of long-term side effects[9]**								
Low/neutral	167 (67.9%)	REF			611 (57.5%)	REF		
High	79 (32.1%)	0.56	0.43-0.72	<0.001	452 (42.5%)	0.87	0.81-0.94	<0.001
Perceptions on STI risk, doxyPEP/PrEP effectiveness and safety								
**Generally being worried contracting an STI[9]**								
Low/neutral	106 (43.1%)	REF			412 (38.8%)	REF		
High	140 (56.9%)	1.15	0.91-1.45	0.245	651 (61.2%)	1.37	1.27-1.48	<0.001
**Finding it awful to contract an STI[9]**								
Low/neutral	129 (52.4%)	REF			479 (45.1%)	REF		
High	117 (47.6%)	0.88	0.70-1.11	0.267	584 (54.9%)	1.18	1.10-1.27	<0.001
**Trust in effectiveness of doxyPEP/PrEP[9]**								
Low/neutral	82 (33.3%)	REF			392 (36.9%)	REF		
High	164 (66.7%)	1.74	1.37-2.24	<0.001	671 (63.1%)	1.50	1.39-1.62	<0.001
**Trust in safety of doxyPEP/PrEP[9]**								
Low/neutral	82 (33.3%)	REF			424 (39.9%)	REF		
High	164 (66.7%)	2.07	1.62-2.65	<0.001	639 (60.1%)	1.56	1.45-1.68	<0.001
**Concerned of AMR due to use[9]**								
Low/neutral	144 (58.5%)	REF			555 (52.2%)	REF		
High	102 (41.5%)	0.61	0.48-0.77	<0.001	508 (47.8%)	0.79	0.74-0.85	<0.001

Abbreviations: CI, confidence interval; HIV, human immunodeficiency virus; OR, odds ratio; PrEP, pre-exposure prophylaxis; PR, prevalence ratio; REF, reference; STI, sexual transmitted infection; AMR, antimicrobial resistance.

1. Data were missing for: country/region of birth (n=8), country/region most connected to (n=7), education level (n=7), employment status (n=7), HIV status and HIV PrEP use (n=10), history of any bacterial STI (n=10), no. of sexual steady partner(s) (n=10), no. of sexual known casual partner(s) (n=11), no. of sexual anonymous casual partner(s) (n=10), condom use steady partner(s) (n=12), condom use known casual partner(s) (n=14), condom use anonymous casual partner(s) (n=13), chemsex (n=7),
sex in combination with alcohol (n=7), sex in combination with other drugs (n=7), injecting drugs during sex (n=7), group sex (n=7), and sex work (n=7).
2. Data were missing for: country/region of birth (n=35), country/region most connected to (n=34), education level (n=32), employment status (n=32), HIV status and HIV PrEP use (n=49), history of any bacterial STI (n=53), no. of sexual steady partner(s) (n=60), no. of sexual known casual partner(s) (n=61), no. of sexual anonymous casual partner(s) (n=58), condom use steady partner(s) (n=60), condom use known casual partner(s) (n=65), condom use anonymous casual partner(s) (n=62), chemsex (n=32),
sex in combination with alcohol (n=32), sex in combination with other drugs (n=32), injecting drugs during sex (n=32), group sex (n=32), sex work (n=32), and reason: to experiment more with sex (n=1).
3. Other is defined as incapacitated, volunteer, retired and studying/in school.

4. In the 6 months before completing the questionnaire.
5. Includes gonorrhoea, chlamydia, lymphogranuloma venereum, and syphilis.
6. Categorised in quantiles.
7. Categorised in quartiles.
8. Includes methamphetamine, gamma-hydroxybutyric acid (GHB)/gamma-butyrolactone (GBL), mephedrone, and ketamine.
9. Dichotomized due to skewed distribution: scores of 1 to 5 were categorized as low/neutral, and scores of 6 and 7 categorized as high.

Note: recent informal use of doxyPEP/PrEP is defined as use in the preceding 6 months.

Supplementary Material S2: STROBE Statement—checklist of items that should be included in reports of observational studies.

	Item No.	Recommendation	Page No.	Relevant text from manuscript
**Title and abstract**	1	(*a*) Indicate the study's design with a commonly used term in the title or the abstract	1	Title page
		(*b*) Provide in the abstract an informative and balanced summary of what was done and what was found	1-2	Title page
**Introduction**				
Background/rationale	2	Explain the scientific background and rationale for the investigation being reported	1	Manuscript
Objectives	3	State specific objectives, including any prespecified hypotheses	1	Manuscript
**Methods**				
Study design	4	Present key elements of study design early in the paper	2	Manuscript
Setting	5	Describe the setting, locations, and relevant dates, including periods of recruitment, exposure, follow-up, and data collection	2-3	Manuscript
Participants	6	(*a*) *Cohort study*—Give the eligibility criteria, and the sources and methods of selection of participants. Describe methods of follow-up<br>*Case-control study*—Give the eligibility criteria, and the sources and methods of case ascertainment and control selection. Give the rationale for the choice of cases and controls<br>*Cross-sectional study*—Give the eligibility criteria, and the sources and methods of selection of participants	1	Manuscript
		(*b*) *Cohort study*—For matched studies, give matching criteria and number of exposed and unexposed<br>*Case-control study*—For matched studies, give matching criteria and the number of controls per case		
Variables	7	Clearly define all outcomes, exposures, predictors, potential confounders, and effect modifiers. Give diagnostic criteria, if applicable	2-4	Manuscript
Data sources/ measurement	8*	For each variable of interest, give sources of data and details of methods of assessment (measurement). Describe comparability of assessment methods if there is more than one group	2-4	Manuscript
Bias	9	Describe any efforts to address potential sources of bias	3-4	Manuscript
Study size	10	Explain how the study size was arrived at	2	Manuscript

Continued on next page

Quantitative variables	11	Explain how quantitative variables were handled in the analyses. If applicable, describe which groupings were chosen and why	2-3	Manuscript
Statistical methods	12	(*a*) Describe all statistical methods, including those used to control for confounding	3-4	Manuscript
		(*b*) Describe any methods used to examine subgroups and interactions	3-4	Manuscript
		(*c*) Explain how missing data were addressed	3-4	Manuscript
		(*d*) *Cohort study*—If applicable, explain how loss to follow-up was addressed<br>*Case-control study*—If applicable, explain how matching of cases and controls was addressed<br>*Cross-sectional study*—If applicable, describe analytical methods taking account of sampling strategy		
		(*e*) Describe any sensitivity analyses	N.A	
**Results**				
Participants	13*	(a) Report numbers of individuals at each stage of study—eg numbers potentially eligible, examined for eligibility, confirmed eligible, included in the study, completing follow-up, and analysed	5	Manuscript
		(b) Give reasons for non-participation at each stage	N.A.	
		(c) Consider use of a flow diagram	N.A.	
Descriptive data	14*	(a) Give characteristics of study participants (eg demographic, clinical, social) and information on exposures and potential confounders	5-8	Manuscript
		(b) Indicate number of participants with missing data for each variable of interest	6-8	Manuscript
		(c) *Cohort study*—Summarise follow-up time (eg, average and total amount)		
Outcome data	15*	*Cohort study*—Report numbers of outcome events or summary measures over time		
		Case-control study—Report numbers in each exposure category, or summary measures of exposure		
		Cross-sectional study—Report numbers of outcome events or summary measures	*5-15*	Manuscript
Main results	16	(*a*) Give unadjusted estimates and, if applicable, confounder-adjusted estimates and their precision (eg, 95% confidence interval). Make clear which confounders were adjusted for and why they were included	5-15	Manuscript
		(*b*) Report category boundaries when continuous variables were categorized	12-15	Manuscript
		(*c*) If relevant, consider translating estimates of relative risk into absolute risk for a meaningful time period		

Continued on next page

Other analyses	17	Report other analyses done—eg analyses of subgroups and interactions, and sensitivity analyses	5-12	Manuscript
**Discussion**				
Key results	18	Summarise key results with reference to study objectives	16-17	Manuscript
Limitations	19	Discuss limitations of the study, taking into account sources of potential bias or imprecision. Discuss both direction and magnitude of any potential bias	17-18	Manuscript
Interpretation	20	Give a cautious overall interpretation of results considering objectives, limitations, multiplicity of analyses, results from similar studies, and other relevant evidence	18	Manuscript
Generalisability	21	Discuss the generalisability (external validity) of the study results	17	Manuscript
**Other information**				
Funding	22	Give the source of funding and the role of the funders for the present study and, if applicable, for the original study on which the present article is based	2	Title page

*Give information separately for cases and controls in case-control studies and, if applicable, for exposed and unexposed groups in cohort and cross-sectional studies.

Note: An Explanation and Elaboration article discusses each checklist item and gives methodological background and published examples of transparent reporting. The STROBE checklist is best used in conjunction with this article (freely available on the Web sites of PLoS Medicine at http://www.plosmedicine.org/, Annals of Internal Medicine at http://www.annals.org/, and Epidemiology at http://www.epidem.com/). Information on the STROBE Initiative is available at www.strobe-statement.org.
